# Characteristics of multidrug-resistant tuberculosis in Namibia

**DOI:** 10.1186/1471-2334-12-385

**Published:** 2012-12-29

**Authors:** Philip M Ricks, Farai Mavhunga, Surbhi Modi, Rosalia Indongo, Abbas Zezai, Lauren A Lambert, Nick DeLuca, Jamie S Krashin, Allyn K Nakashima, Timothy H Holtz

**Affiliations:** 1 Centers for Disease Control and Prevention, Atlanta, GA, USA; 2 Ministry of Health and Social Services, Windhoek, Namibia; 3 KNCV Tuberculosis Foundation, Windhoek, Namibia; 4 Centers for Disease Control and Prevention, Windhoek, Namibia

## Abstract

**Background:**

To describe the epidemiology and possible risk factors for the development of multidrug-resistant tuberculosis (MDR-TB) in Namibia.

**Methods:**

Using medical records and patient questionnaires, we conducted a case-control study among patients diagnosed with TB between January 2007 and March 2009. Cases were defined as patients with laboratory-confirmed MDR-TB; controls had laboratory-confirmed drug-susceptible TB or were being treated with WHO Category I or Category II treatment regimens.

**Results:**

We enrolled 117 MDR-TB cases and 251 TB controls, of which 100% and 2% were laboratory-confirmed, respectively. Among cases, 97% (113/117) had been treated for TB before the current episode compared with 46% (115/251) of controls (odds ratio [OR] 28.7, 95% confidence interval [CI] 10.3–80.5). Cases were significantly more likely to have been previously hospitalized (OR 1.9, 95% CI 1.1–3.5) and to have had a household member with MDR-TB (OR 5.1, 95% CI 2.1–12.5). These associations remained significant when separately controlled for being currently hospitalized or HIV-infection.

**Conclusions:**

MDR-TB was associated with previous treatment for TB, previous hospitalization, and having had a household member with MDR-TB, suggesting that TB control practices have been inadequate. Strengthening basic TB control practices, including expanding laboratory confirmation, directly observed therapy, and infection control, are critical to the prevention of MDR-TB.

## Background

Multidrug-resistant tuberculosis (MDR-TB) has become a major public health problem and obstacle to global TB control [1,2]. MDR-TB is associated with higher case fatality rates, especially among HIV-infected patients [3,4], and is much more difficult and costly to treat than drug-susceptible TB [5]. The worldwide burden of MDR-TB has been growing, and in 2008, there were 440,000 estimated new cases of MDR-TB, or 3.6% of all incident TB cases, compared to 273,000 estimated new cases (3.2% of incident TB cases) in 2000 [5-7]. However, these estimates may not accurately represent the true global burden of MDR-TB as they are based on surveillance reports from only 114 countries, of which only 10 are sub-Saharan countries with recent or complete national data [5].

Despite the lack of comprehensive surveillance data from Africa, MDR-TB has been recognized as an emerging public health concern. In South Africa, clusters of MDR-TB cases have been documented in institutional and community settings and among HIV-infected patients [3,8-12]. Epidemiologic investigations of these and other outbreaks have demonstrated that previous treatment for TB and transmission in institutional and community settings are important risk factors in MDR-TB development [10-15].

Since 2007, the National TB Control Program (NTCP) in Namibia has documented an increase in drug-resistant TB (DR-TB) [5,16]. However, there are few population-level data available to evaluate the prevalence of MDR-TB in Namibia. Namibia has one of the highest TB incidence rates in the world, estimated at 665 new cases per 100,000 persons in 2008 [17]. Among incident TB cases, 59% are estimated to be co-infected with HIV [17]. In 2008, 201 cases of MDR-TB were reported to the NTCP, and the estimated prevalence of MDR-TB was 3.8% among new smear-positive TB cases and 16.5% among previously treated TB cases [17]. However, drug-susceptibility testing (DST) is not routinely performed, making the reported prevalence of MDR-TB in Namibia an underestimate of the true burden.

Data about the relative contributions of specific risk factors to the development of MDR-TB in Namibia are also limited. In late 2008, an investigation of only 34 DR-TB patients by the NTCP and the U.S. Centers for Disease Control and Prevention (CDC) found clinical evidence suggesting acquired TB drug resistance related to incomplete TB treatment in the community and primary transmission of DR-TB in the hospital settings (NTCP, unpublished data). In this report, we describe a more extensive epidemiologic investigation of MDR-TB in Namibia, including possible risk factors associated with MDR-TB.

## Methods

### Ethics statement

This investigation was requested by Namibia's Ministry of Health and Social Services (MOHSS) and was deemed to be a response to an urgent public health problem. The investigation consisted of two parts, data abstraction from medical records and patient interviews. The data abstraction component of the investigation was determined to be an outbreak response; CDC and the Namibian MOHSS do not require human subjects consent for review of routine medical records during outbreak investigations. The patient interview portion of the investigation was conducted by staff from the Namibian MOHSS. Patients' verbal informed consent was obtained prior to the interview, but was not required to be documented. The interviews were conducted in the context of TB education and consent was obtained while the interviewer determined which language the patient felt most comfortable speaking. Medical records data and patient interview data were linked by patient ID, which was removed prior to analysis, which was done anonymously.

### Study design, site selection and participants

We conducted a case-control study among patients diagnosed with TB between January 1, 2007 and March 31, 2009 in nine major cities in Namibia (Table 1). Study participants were selected from inpatient and outpatient departments of the main hospital providing TB services in each city, and their respective local community-based DOT clinics. These nine hospitals included seven of eight NTCP-designated regional DR-TB treatment centers.

**Table 1:** Distribution of demographic and treatment characteristics of cases and controls

		**Cases (N = 117)**		**Controls (N = 251)**		**p value**^**a**^
		**n (%) or Mean (SD)**		**n (%) or Mean (SD)**		
Education, years of schooling		7.5 (3.8)		7.4 (3.6)		0.87
Household size, number of people		6.3 (4.2)		5.8 (4.8)		0.31
Age (yrs.)	<21	11	(9)	25	(10)	0.89
	21-29	23	(20)	52	(21)	
	30-39	41	(35)	95	(38)	
	40-49	27	(23)	46	(18)	
	50-64	12	(10)	23	(9)	
	65+	3	(3)	10	(4)	
Male		65	(56)	139	(55)	0.93
Married/partnered		33	(28)	63	(25)	0.35
Born in Namibia		111	(95)	242	(96)	1.00
Treatment city	Windhoek	36	(31)	79	(31)	<.0001
	Walvis Bay	23	(19)	28	(11)	
	Oshakati	23	(19)	49	(20)	
	Onandjokwe	7	(6)	26	(10)	
	Grootfontein	8	(7)	22	(9)	
	Rundu	7	(6)	10	(4)	
	Luderitz	6	(5)	10	(4)	
	Otijiwarongo	4	(4)	12	(5)	
	Keetmanshoop	3	(3)	15	(6)	
Currently hospitalized		97	(83)	77	(31)	<.0001
Current treatment regimen	Category I	0	(0)	157	(63)	<.0001
	Category II	1	(1)	94	(37)	
	Category IV	116	(99)	0	(0)	
Documented HIV testing		108	(92)	212	(84)	0.04
HIV- infected^b^		55	(51)	141	(67)	0.007
Documented CD4 test result^c^		24	(44)	56	(40)	0.76
Receiving ART ^c^		32	(58)	59	(42)	0.04
Documented DST result	ALL	117	(100)	4	(2)	<.0001
	MDR-TB	112	(96)	0	(0)	
	XDR-TB^d^	5	(4)	0	(0)	
Previous TB treatment		113	(97)	115	(46)	<.0001
Previous Category IV treatment		35	(30)	0	(0)	<.0001

^a^ p values are for known outcome values.

^b^ among those with a documented HIV test.

^c^ among those HIV-infected.

^d^ defined as a *Mycobacterium tuberculosis* isolate with resistance to isoniazid and rifampin,

ofloxacin, and one of three injectable second-line drugs.

For our study, we defined cases as patients with a laboratory-confirmed diagnosis of MDR-TB or extensively drug-resistant TB (XDR-TB). MDR-TB was defined as a *Mycobacterium tuberculosis* isolate with resistance to at least isoniazid and rifampin. XDR-TB was defined as a *Mycobacterium tuberculosis* isolate with resistance to isoniazid and rifampin, ofloxacin, and one of three injectable second-line drugs [2].

We defined controls as patients if (1) they had laboratory-confirmation of a TB isolate susceptible to isoniazid, rifampin, ethambutol, and streptomycin (DST not done on pyrazinamide); or (2) DST was not performed but they were receiving Category I or Category II treatment regimens in accordance with WHO and NTCP guidelines [16,18,19].

### NTCP TB treatment guidelines

In Namibia, TB culture and DST are reserved for TB patients who have been previously treated or are suspected of having DR-TB. Patients with confirmed MDR-TB are required to receive inpatient treatment until sputum culture conversion and can then complete their treatment at an outpatient directly observed therapy (DOT) facility [16,18,20]. Patients with presumed drug-susceptible TB receive treatment through community-based outpatient DOT clinics, unless complications require hospitalization. At the time of this investigation, TB treatment regimens in Namibia were based on 2003 WHO guidelines, defined as follows: [18,19].

Category I – Initial phase of two months of isoniazid (H), rifampin (R), pyrazinamide (Z), ethambutol (E) daily followed by continuation phase of four months RH, for new patients with any form of tuberculosis.

Category II – Initial phase of two months HRZE daily and streptomycin (S) on weekdays, followed by one month of HRZE daily, followed by continuation phase of five months of RHE daily. To be used for relapse or return after default of either Category I or Category II regimens, treatment failure, or recurrent tuberculosis.

Category IV – Specially designed standardized or individualized regimens for chronic (still sputum-positive after supervised re-treatment); proven or suspected MDR-TB patients.

### Patient enrollment

Cases and controls were selected using convenience sampling. For cases, we attempted to enroll all hospitalized MDR-TB patients at the study hospitals and all MDR-TB patients being treated on an outpatient basis if they came to the hospital's outpatient clinic or the local DOT clinic during our visit.

For controls, we attempted to enroll all patients who met our study's definition of a control if they visited the hospital outpatient clinic or local DOT clinic to receive TB treatment on the days of our visit. We also selected a convenience sample of hospitalized TB patients who met our control definition.

### Data collection

Data were obtained through two different methods: 1) data abstraction from medical records, and 2) patient interviews. For the medical data, we abstracted data from TB treatment cards, patient hospital and TB clinic charts, and patient health passports (a small booklet containing a person′s medical history) of cases and controls using a modified data collection form from previous MDR-TB investigations [21,22]. We collected basic demographic information; information on previous and current TB episodes; HIV infection and treatment; and TB-related laboratory data. Study patients were classified as previously treated for TB if there was evidence of treatment for a previous TB episode in their medical record.

MOHSS health care personnel interviewed cases and controls in their local language, using a structured questionnaire, to collect additional information about socioeconomic status and possible risk factors for DR-TB, such as information about previous episodes of TB, adherence to DOT, and contact with TB and MDR-TB patients. MOHSS shared the interview data with the team conducting the medical data abstraction, and the datasets were linked by the patient TB registry number. The patient TB registry number was removed from all electronic datasets once the linkage was completed and all hard copies of data abstraction and interview forms were given to Namibia′s MOHHS for safekeeping and storage.

### Data management and data analysis

To detect significant differences in the distributions of descriptive quantitative variables between cases and controls, we used the chi-square statistic or Fisher's exact, for cell size <=5, for dichotomous and categorical variables and t-tests for continuous and ordinal variables. A two-sided univariate analysis was used to evaluate the relationship between potential risk factors and MDR-TB. For those variables significantly associated with MDR-TB, at the level of p<0.05, an additional two-sided univariate analysis was performed, controlling for those descriptive variables that showed the most significant difference, p<0.01, in distributions between cases and controls. Two sub-analyses performed: one using only Category I treatment controls and one using only Category II treatment controls. All data were analyzed using the statistical program SAS 9.1 (SAS Institute, Cary, NC, USA). Statistical significance was considered at an alpha of <0.05.

## Results

### Patient characteristics

We reviewed medical records of 368 patients, of whom 117 (32%) were MDR-TB cases and 251 (68%) were TB controls. All 117 cases (100%) and 4 (2%) controls were confirmed by DST. Having a DST performed was significantly associated with previous treatment (p<0.0001), reporting a household member with MDR-TB (p=0.0004), and previous hospitalization (p=0.018). Interviews were conducted with the 106 (91%) cases and 244 (97%) controls available when study staff was on-site. Demographic characteristics were similar between cases and controls: mean age was roughly 36 years, approximately 55% were male, more than 90% were born in Namibia, and over 60% were treated in one of three large cities (Table 1). Patient knowledge about TB was limited: 75 (64%) cases and 167 (67%) controls did not know that TB was spread through person-to-person transmission (p =0.83).

MDR-TB cases were significantly more likely to have been previously treated for TB, although previous treatment was also common among controls, 113 (97%) and 115 (46%), respectively; p < 0.0001. Thirty-five (30%) cases had been previously treated with a Category IV regimen. Among those who were previously treated for TB, 77 (68%) of cases and 80 (70%) of controls had a TB treatment outcome of default, failure, or unknown outcome for the most recent previous TB treatment episode (p=0.82).

Documentation of HIV testing was high among both cases and controls, at 108 (92%) and 212 (84%), respectively (p = 0.04). Of those who were tested, 55 (51%) of cases were HIV-infected compared to 141 (67%) of controls (p = 0.007). Of those who were HIV-infected, 32 (58%) of cases and 59 (42%) of controls were on antiretroviral therapy (ART) (p=0.04). Among HIV-infected patients who were not yet on ART, 6/23 (26%) cases and 36/82 (44%) of controls could be considered for ART based on their recorded CD4 count and clinical stage, according to Namibian guidelines [18,23].

### Possible risk factors associated with being an MDR-TB case

In a univariate analysis, having a documented previous TB episode was significantly associated with being an MDR-TB case (odds ratio [OR] = 28.7, 95% confidence interval [CI] 10.3–80.5). Having had a household member with MDR-TB (OR = 5.1, 95% CI 2.1–12.5), documentation of HIV testing (OR = 2.2, 95% CI 1.0–4.7), previous hospitalization (OR = 1.9, 95% CI 1.1–3.5), and receiving ART (OR = 2.3, 95% CI 1.1–4.6), were significantly associated with being a case (Table 2). HIV infection was inversely associated with being a case (OR = 0.5, 95% CI 0.3–0.9).

**Table 2:** Association between independent demographic and clinical characteristics and MDR-TB case

		**Cases**^**a**^		**Controls**^**a**^		**OR (95% CI)**
		**n**	**(%)**	**n**	**(%)**	
Type of housing	Mud	13	(12)	7	(3)	4.6 (1.8 – 11.9)
	All other types	94	(88)	234	(97)	1.0
Previous TB treatment	Yes	113	(97)	115	(50)	28.7 (10.3 – 80.5)
	No	4	(3)	117	(50)	1.0
Site of TB ^b^	Pulmonary	115	(98)	183	(93)	4.4 (0.9 – 19.7) ^c^
	With extra-pulmonary	2	(2)	14	(7)	1.0
Currently hospitalized	Yes	97	(85)	77	(31)	12.8 (6.9 – 24.0)
	No	17	(15)	173	(69)	1.0
Previously hospitalized ^b, d^	Yes	71	(70)	54	(55)	1.9 (1.1 – 3.5)
	No	30	(30)	44	(45)	1.0
Household TB contact	Yes	64	(62)	130	(55)	1.4 (0.8 – 2.2)
	No	39	(38)	108	(45)	1.0
Household MDR-TB contact ^b^	Yes	16	(21)	8	(5)	5.1 (2.1 – 12.5)
	No	60	(79)	152	(95)	1.0
Household respiratory death^e^	Yes	32	(31)	57	(24)	1.4 (0.8 – 2.3)
	No	71	(69)	176	(76)	1.0
Documented HIV testing	Yes	108	(92)	212	(84)	2.2 (1.0 – 4.7)
	No	9	(8)	39	(16)	1.0
HIV infection ^b^	Positive	55	(51)	141	(67)	0.5 (0.3 – 0.9)
	Negative	53	(49)	71	(33)	1.0

^a^ Different denominators due to ″unknown″ and missing data.

^b^ =>10% of responses ″unknown″ or missing data.

^c^ Fisher′s exact test p=0.036.

^d^ Previously hospitalized for any reason.

^e^ Household member death from TB/respiratory illness.

In a univariate analysis adjusting for current hospitalization status, previous hospitalization and documented HIV testing were no longer significantly associated, while HIV infection remained negatively associated with being an MDR-TB case (Table 3). The crude odds ratios for all possible risk factors associated with being an MDR-TB case, when adjusted for HIV infection, remained significant (Table 3). A documented previous TB episode continued to be the strongest possible risk factor for MDR-TB status (OR = 23.2, 95% CI 8.4–64.4). In the sub-analyses comparing all MDR-TB cases to the subset of controls receiving Category I treatment, HIV infection no longer had a negative association among the six unadjusted possible risk factors associated with being an MDR-TB case (Table 4). In comparing all MDR-TB cases to the subset of controls receiving Category II treatment, HIV infection remained negatively associated and having had a household member with MDR-TB remained positively associated with being an MDR-TB case.

**Table 3:** Association between selected characteristics and MDR-TB case, adjusted for current hospitalization status and HIV infection

			**Adjusted for current**		**Adjusted for**	
	**Crude association**		**hospitalization status**		**HIV infection**	
	**OR**	**(95% CI)**	**AOR**	**(95% CI)**	**AOR**	**(95% CI)**
Previous TB treatment (N=349)	28.7	(10.3 – 80.5)	13.5	(4.5 – 40.9)	23.2	(8.4 – 64.4)
Currently hospitalized (N=364)	12.8	(6.9 – 24.0)	n/a	n/a	11.5	(6.3 – 21.3)
Previously hospitalized, any reason (N=199)	1.9	(1.1 – 3.5)	1.3 ^a^	(0.6 – 2.7)	1.9	(1.0 – 3.5)
Household MDR-TB contact (N=236)	5.1	(2.1 – 12.5)	3.8	(1.3 – 10.8)	5.3	(2.0 – 13.8)
Documented HIV testing (N=368)	2.2	(1.0 – 4.7)	1.4 ^a^	(0.6 – 3.7)	n/a	n/a
HIV infection (N=320), pos. vs. neg.	0.5	(0.3 – 0.9)	0.5	(0.3 – 0.8)	n/a	n/a
Receiving ART (N=169)	2.3	(1.1 – 4.6)	2.7	(1.2 – 6.0)	2.3	(1.1 – 4.6)

^a^ ORs are no longer statistically significant when adjusted, p> 0.05.

**Table 4:** Association between selected characteristics and MDR-TB case, using two different control subgroups

			**Using only Category I**		**Using only Category II**	
	**Crude Association**		**Controls (N=157)**		**Controls (N=94)**	
	**OR**	**(95% CI)**	**OR**	**(95% CI)**	**AOR**	**(95% CI)**
Previous TB episode	28.7	(10.3 – 80.5)	110.9	(37.7– 326.8)	2.3 ^a^	(0.6 – 8.0)
Previously hospitalized, any reason	1.9	(1.1 – 3.5)	3.4	(1.7 – 6.7)	0.8 ^a^	(0.4 –1.9)
Household MDR-TB contact	5.1	(2.1 – 12.5)	6.1	(1.9 – 19.2)	4.0	(1.3 – 12.7)
Documented HIV testing	2.2	(1.0 – 4.7)	2.4	(1.1 – 5.3)	1.9 ^a^	(0.8 – 4.7)
HIV infection, pos. vs. neg.	0.5	(0.3 – 0.9)	0.6 ^a^	(0.4 – 1.1)	0.4	(0.2 – 0.7)
Receiving ART	2.3	(1.1 – 4.6)	2.8	(1.3 – 6.1)	1.7 ^a^	(0.7 – 3.8)

^a^ ORs are not statistically significant, p< 0.05.

## Discussion

Detecting the emergence and controlling the spread of MDR-TB begins with its timely diagnosis. However, only 3% of re-treatment controls had a documented DST, despite NTCP′s recommendation that all re-treatment patients receive DST. While recognizing limited resources for DST and the high percentage of TB retreatment, Namibia needs to improve diagnosis of DR TB. This will necessitate impressing upon health care personnel the need to comply with NTCP DST guidelines.

Compared to controls, we found MDR-TB cases were 28 times more likely to have been treated for a previous TB episode, and that among those previously treated, the most recent outcome was failure, default, or unknown for roughly 70% of both cases and control. The association between previous TB treatment and MDR-TB has been noted in investigations in other countries [13-15,24,25], while more recent studies have observed that it is previous treatment failure that is significantly associated with MDR-TB [3,25] Our findings underscore the need to improve basic TB control practices to ensure that all TB patients adhere to and complete TB treatment. TB has been recognized as a significant public health problem in Namibia since its independence in 1990 and the NTCP has continually revised its TB control guidelines, in order incorporate updated WHO recommendations and address DR-TB [16,18,19,26]. However, considerable difficulties exist in providing adequate DOT in a vast but sparsely populated country with high TB prevalence and shortages of trained health care workers [17]. As in other sub-Sahara African countries, the NTCP has introduced community DOT treatment, so that patients in remote areas can easily access DOT [27-29]. These and other interventions need to be strengthened to improve basic DOT coverage and prevent continued emergence of drug-resistant strains in Namibia.

Evidence of transmission of MDR-TB within households and in the community in the current study is not surprising and has also been documented in neighboring South Africa [11,12]. In our investigation, nearly two-thirds of patients being treated for TB were unaware of how TB is spread, making it unlikely that these patients or their families were aware of measures that could prevent the spread of TB. These findings suggest a need for increased TB education for cases and their household members. In the current study, MDR-TB cases were almost twice as likely to have been previously hospitalized, and nosocomial spread of MDR-TB and institutional outbreaks of XDR-TB have been well-documented in sub-Saharan Africa and elsewhere [10,13,30]. Although Namibia developed new infection control guidelines in 2008 and WHO recently revised its policy on TB infection [20,31], TB infection control measures have not been widely implemented in most Namibian facilities. It is essential for countries such as Namibia to implement effective infection control practices, especially in institutional settings.

Like many countries in sub-Saharan Africa, Namibia has experienced an increase in TB incidence, from 322 per 100,000 persons in 1990 to a peak of 817 per 100,000 persons in 2004, largely related to the growing HIV epidemic. There is conflicting evidence as to whether HIV is an independent risk factor for DR-TB [24,25,32,33]. In our investigation, we found that HIV infection was negatively associated with MDR-TB, i.e. HIV infection was ″protective″ against MDR-TB. As suggested by other studies, this may have been due to HIV- infected persons dying at a higher rate than HIV- uninfected person, such that HIV-infected persons were less likely to live to the point of either developing MDR-TB, or to die before being diagnosed with MDR-TB. The higher death rate among HIV- infected persons is possibly caused by the combination of 1) poor treatment outcomes, due to malabsoprtion of anti-TB drugs or lower rates of adherence, and 2) a greater likelihood of being exposed to MDR-TB patients during medical visits or hospitalizations [3,34-39]. With CD4 count information documented for less than half of HIV-infected cases and controls, improved linkages to HIV care and treatment services and earlier initiation of ART are critical. WHO recently revised its treatment recommendations for HIV-infected TB patients to initiate ART earlier, regardless of CD4 count, and this may be especially important for HIV-infected patients with MDR-TB [40].

Although our investigation showed associations between various characteristics and MDR-TB, assessing these as risk factors is constrained by the limitations of our investigation. First, because of Namibia's resource limitation and the urgent nature of our investigation, DST was performed almost exclusively (96%) on retreatment patients, making retreatment necessary for obtaining an MDR-TB diagnosis. Thus, it was not possible to fully assess the extent of primary MDR-TB transmission and the lack of previous treatment outcome data did not allow us to assess this effect. Second, few controls had a documented DST result, potentially leading to misclassification bias. Generally, this type of misclassification would bias the associations towards the null, suggesting that the associations reported in this investigation are true associations. However, as the major associations were also associated with having a DST, the effect of this misclassification might have a more complex effect. We performed two sub-analyses that restricted the control groups to either CAT I, of which 28/142 (20%) were previous treated for TB or CAT II, of which 87/94 (93%) were previously treated. These analyses suggest that CAT II controls may have been more likely to be misclassified as compared to CAT I controls, for which 5 of the 6 crude associations remained significant. Third, because NTCP guidelines recommend inpatient treatment for patients with DR-TB but not drug-susceptible TB, patients selected from inpatient facilities were more likely to be MDR-TB cases. Documentation was more extensive for inpatients, which could have resulted in an information bias for MDR-TB cases. Although hospitalization status confounds the magnitude of the crude associations, it does not change their significance or direction. Lastly, we were unable to fully assess the temporality of contact with patients with TB or MDR-TB and our study cannot definitively establish nosocomial or household transmission. Additional investigations in Namibia are needed to adequately describe the amount of primary and acquired MDR-TB.

## Conclusion

In summary, we found that previous TB treatment, previous hospitalization, and household contact with an MDR-TB case were associated with MDR-TB in Namibia. Our findings reinforce the importance of maintaining strong overall TB control measures, to ensure adherence to diagnostic protocols, that all TB patients complete an appropriate treatment regimen through DOT programs, and that appropriate household and institutional infection control measures are observed. This has become even more critical, in light of the high prevalence of HIV-infection in Namibia, which may decrease adherence and/or the effectiveness of TB treatment and increase the risk of exposure to MDR-TB due to HIV-related hospitalizations. Finally, enhancing patient education is critical in helping patients and their families understand the importance of treatment adherence and implementing measures to prevent the spread of TB within the household and the community.

## Competing interests

The authors declare that they have no competing interests.

## Authors' contributions

PMR participated in study design, study logistics, data collection, data management, data entry, performed all data analysis and drafted the drafted manuscript. FM participated in study design, study logistics, data collection, and critical revision of manuscript. RI participated in study design, study logistics, data collection, and editing of manuscript. SM participated in study design, study logistics, data collection, data management, data entry, and the editing and preparation of manuscript. AZ participated in study design, data collection, and editing of manuscript. LAL participated in study logistics, data collection, data management, data entry, and editing of manuscript. ND participated in study design, study logistics, and editing of manuscript. JSK participated in study logistics, data collection, data management, data entry, and editing of manuscript. AKN participated in study design, and editing and preparation of manuscript. THH participated in study design, study logistics, data collection, and editing and preparation of manuscript. All authors read and approved the final manuscript.

## Disclaimer

The findings and conclusions in this report are those of the authors and do not necessarily represent the views of U.S. Centers for Disease Control and Prevention.

## Pre-publication history

The pre-publication history for this paper can be accessed here:

http://www.biomedcentral.com/1471-2334/12/385/prepub
